# Anti-3-hydroxy-3-methylglutaryl coenzyme A reductase immune-mediated necrotizing myopathy with a CD68+ lymphohistiocytic cutaneous eruption

**DOI:** 10.1016/j.jdcr.2025.01.027

**Published:** 2025-02-22

**Authors:** Yeojin Park, Cyrelle F. Finan, Hillary Elwood, Nikifor K. Konstantinov

**Affiliations:** a Department of Dermatology, University of New Mexico School of Medicine, Albuquerque, New Mexico; b Division of Dermatology, Children’s National Medical Center, Washington, District of Columbia; c TriCore Reference Laboratories, Department of Dermatopathology, University of New Mexico, Albuquerque, New Mexico

**Keywords:** autoantibody, autoimmune disease, case reports (general dermatology), connective tissue disease, corticosteroids, dermatomyositis, dermatopathology, drug reactions, histiocytosis (x, etc), immunodermatology, immunology, immunopathology, inflammation/inflammatory, pathology (inflammatory diseases)

## Introduction

Anti-3-hydroxy-3-methylglutaryl coenzyme A reductase immune-mediated necrotizing myopathy (anti-HMGCR IMNM) is a rare form of myositis associated with statin use. It typically presents with an acute onset and has a poor prognosis. Early recognition and prompt treatment with intravenous immunoglobulin (IVIG) and systemic corticosteroids are crucial for managing this condition. Cutaneous involvement is rare, but dermatomyositis-like features have been reported.^1^, ^2^, ^3^ We report a case with cutaneous features resembling dermatomyositis and skin pathology demonstrating an interstitial lymphohistiocytic infiltrate.

## Case report

**Fig 1:**
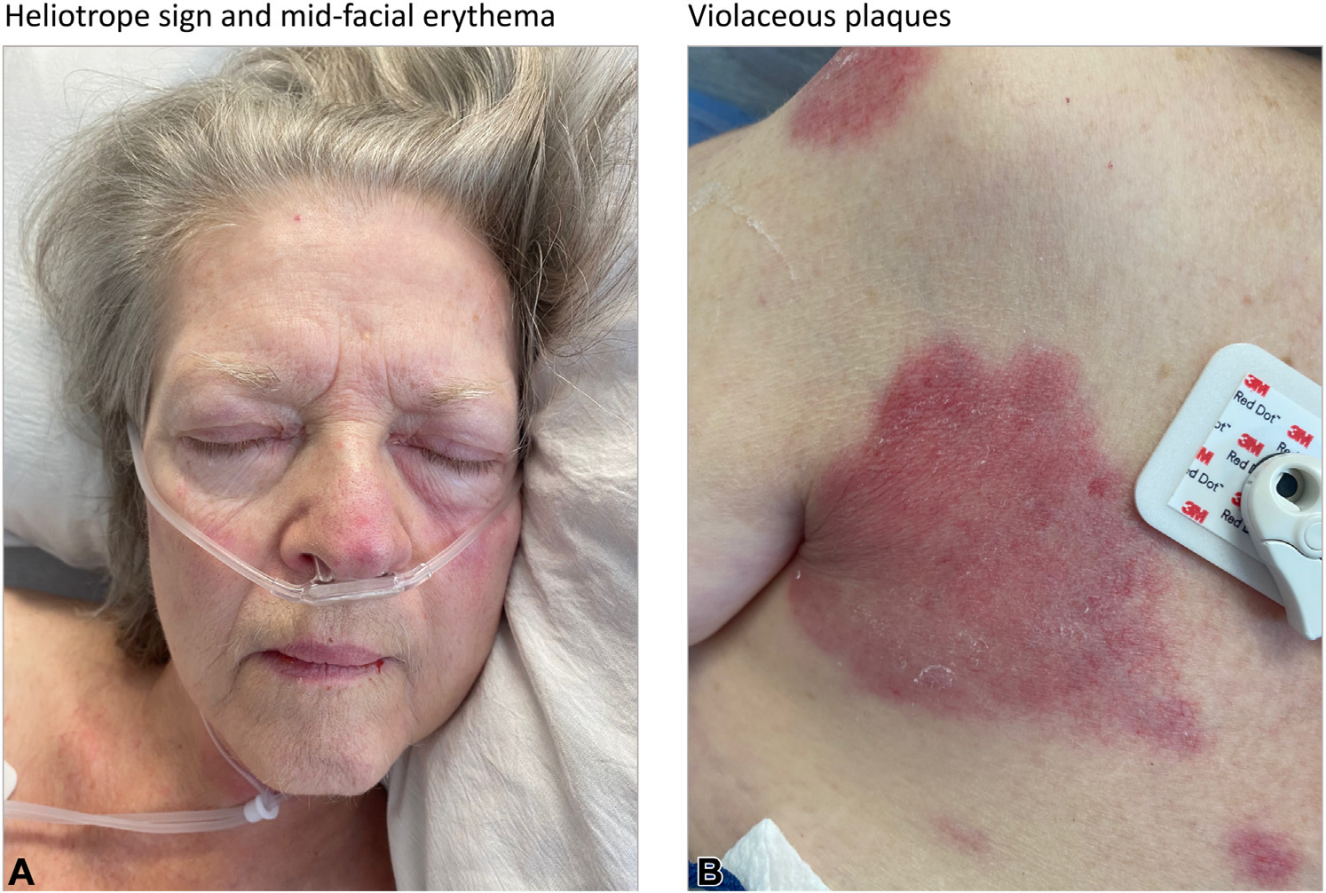
Clinical features of the dermatomyositis-like cutaneous eruption. **A,** Violaceous patches on bilateral cheeks and eyelids, consistent with heliotrope sign and midfacial erythema. **B,** Violaceous plaque of the right chest and shoulder.

**Fig 2:**
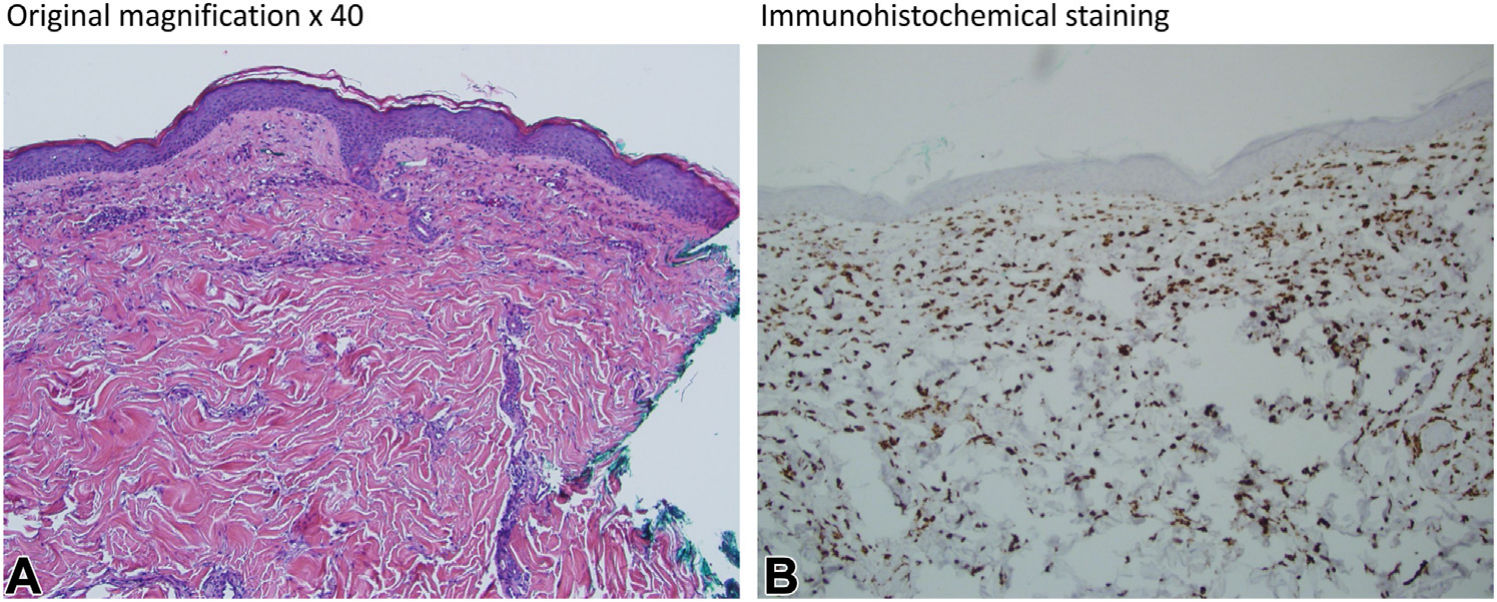
Histopathology of right chest plaque. **A,** Dermal perivascular to interstitial lymphohistiocytic infiltrate (×40, hematoxylin & eosin stain, H&E). **B,** CD68+ immunohistochemistry stain confirming the presence of histiocytes.

A 72-year-old woman with a history of dementia, and hyperlipidemia on atorvastatin, was admitted to the hospital with fatigue and a 2-week history of progressive dysphagia, rash, and weakness in the upper and lower bilateral extremities. The patient was not on any new medications and had been on long-standing statin therapy (on atorvastatin transitioned from simvastatin 18 months prior). The skin examination was notable for a mild heliotrope sign, midfacial erythema, and violaceous-colored plaques on her chest, shoulder, and anterior neck (Fig 1). Laboratory results included a negative antinuclear antibody, an elevated creatinine kinase level (5130 u/L), and an elevated anti-HMGCR > 200 units. The punch biopsy of the right chest revealed a dermal perivascular to interstitial lymphohistiocytic infiltrate, resembling a subtle interstitial granulomatous dermatitis, without increase in dermal mucin or interface change. A CD68 stain confirmed the presence of a histiocytic component (Fig 2). Left thigh muscle biopsy revealed a necrotizing myopathy with minimal inflammation.

The patient’s diagnosis was consistent with anti-HMGCR IMNM. After discontinuing her atorvastatin, she was treated initially with intravenous methylprednisolone 500 mg/day for 3 days and IVIG 2 g/kg given over 2 days. Her myositis progressed and she developed respiratory failure with left lower lobe pneumonia. She expired 11 days after admission.

## Discussion

Anti-HMGCR IMNM is a rare myositis induced by statins or food and dietary substances in which statins are present (ie, Oyster mushroom, red yeast rice, and Pu-erh tea).^3^, ^4^, ^5^ IMNM is characterized by proximal muscle weakness, elevated creatinine kinase level (more than 2000 IU/L in 90% of cases), and abundant necrosis of muscle fibers with minimal inflammatory infiltrate on muscle biopsy.^5^ In anti-HMGCR myopathy, most of the inflammatory infiltrate in the muscle histology is composed of CD68+ macrophages. There is scant literature on skin biopsy findings, with one previously documented case also showing superficial and deep dermal interstitial lymphohistiocytic infiltrate^6^ and another showing dermal perivascular lymphohistiocytic infiltrate.^7^ CD68 is a macrophage cell marker and patients with IMNM exhibit a strong type 1 helper T cell and activated macrophage M1 response, leading to elevated interferon-γ, tumor necrosis factor-α, interleukin-12, and signal transducer and activator of transcription 1 levels in the muscle tissue causing damage. Immunohistochemical profile comparing IMNM and non-IMNM shows significantly more CD68+ macrophages, CD4+ cells, and CD8+ T cells.^8^

Anti-HMGCR IMNM is characterized by its acute onset with poor prognosis.^5^ In several studies, skin involvement ranged from 15% to 44% of patients.^1^ The most common cutaneous manifestations reported include Raynaud’s phenomenon, Gottron’s sign, heliotrope rash, V-sign, and periungual lesions. Life-threatening dysphagia was often reported, among other extramuscular symptoms.^5^

Affected patients range in age, between 40 and 60 years, with female predominance.^2^ Anti-HMGCR IMNM occurs in about 2-3/100,000 patients treated with a statin. Timing of skin manifestations can vary after statin exposure.^2^ Patients with dermatomyositis-like rashes had a significantly earlier mean age of onset with a shorter mean disease duration.^1^ Statin exposure is observed in up to two-thirds of anti-HMGCR antibody positive IMNM. The patient had been intermittently taking simvastatin for nearly 2 decades before transitioning to atorvastatin 18 months prior to disease development. While atorvastatin is found to have higher risk for this condition than other statins,^9^ the patients had exposure to both. Since the range of exposure from statin to onset can range from weeks to years without a definitive range,^10^ long-term exposure to statins is likely required to trigger the necrotizing myopathy. Early recognition is significantly associated with early remission and adequate maintenance.^2^ Initial therapy should include systemic corticosteroids often in combination with IVIG.

The case highlights a patient with anti-HMGCR antibody positive IMNM with dermatomyositis-like cutaneous eruption. Atypical violaceous cutaneous eruptions, as noted on the patient’s chest and shoulder, may also be seen. It is important to note that these patients may not have the classic histopathologic findings of dermatomyositis, and biopsy may show an interstitial CD68+ histiocytic infiltrate. Although the presence of the CD68+ infiltrate in the skin may support this drug-induced myopathy, further studies are needed. Interstitial granulomatous dermatitis or a drug eruption may also resemble the histologic diagnosis, but the synchronous development of the facial and truncal rashes favored a manifestation of this immune-mediated necrotizing myopathy. Anti-HMGCR antibody screening should be considered in the case of myositis with dermatomyositis-like features particularly in the case of statin exposure.

## Conflicts of interest

None disclosed.
